# Urinary protein semi-quantification in immunochemotherapy for bladder cancer: markers of clinical outcomes

**DOI:** 10.3389/fimmu.2026.1929760

**Published:** 2026-08-10

**Authors:** Houyuan Chen, Yuda Lin, Zihan Xue, Yunkai Qie, Zhouliang Wu, Kangkang Liu, Shiwang Huang, Kaipeng Jia, Shaobo Yang, Zhe Zhang, Peng Li, Xiaohua Li, Liwei Liu, Chong Shen, Hailong Hu

**Affiliations:** 1 Department of Urology, The Second Hospital of Tianjin Medical University, Tianjin, China; 2 Tianjin Key Laboratory of Urology, Tianjin Institute of Urology, The Second Hospital of Tianjin Medical University, Tianjin, China

**Keywords:** bladder cancer, clinical outcomes, immunochemotherapy, nab-paclitaxel, PD-1 immune checkpoint inhibitors, urinary protein semi-quantification

## Abstract

**Objective:**

PD-1 based immunochemotherapy has improved bladder cancer (BC) outcomes, yet individual responses remain heterogeneous. Low-cost, non-invasive predictive biomarkers are urgently needed. Urinary protein semi-quantification (UPSQ) correlates with BC tumor load, but its predictive value during PD-1 based immunochemotherapy remains unclear. We conducted a *post-hoc* analysis of the TRUCE-01 (NCT04730219) and TRUCE-02 (NCT04730232) trials to explore the association between UPSQ and clinical outcomes.

**Methods:**

104 patients received 3 cycles of tislelizumab plus nab-paclitaxel. UPSQ at baseline (UP-B) and 60 ± 7 days (UP-60) after treatment initiation, urinary red blood cell (URBC) and urinary white blood cell (UWBC) were dynamically detected. Dynamic indices (UPDV score, UPV risk stratification) were defined to evaluate UPSQ changes. The primary endpoint was clinical complete response (cCR), and the secondary endpoints included overall survival (OS), cancer-specific survival (CSS), and events-free survival (EFS). Bulk RNA-seq transcriptomic analysis was performed in 22 MIBC patients.

**Results:**

UPSQ decreased significantly at 60 days in the overall cohort and subgroups (all p < 0.05). Baseline URBC and UWBC were positively correlated with UPSQ, while tumor size was positively associated with UPSQ in MIBC patients. In regression analyses, several indicators reached nominal significance (*unadjusted p* < 0.05), but failed to remain significant (*adjusted p* > 0.05) after Benjamini-Hochberg correction due to limited sample size. Low UPV risk was correlated with a significantly higher cCR rate in both MIBC (OR 12.5, *unadjusted p* = 0.003, *adjusted p* = 0.055) and VHR-NMIBC (OR 3.47, *unadjusted p* = 0.031, *adjusted p* = 0.177) subgroups. UP-B grade showed a trend toward an inverse association with cCR (OR 0.48, *unadjusted p* = 0.028, *adjusted p* = 0.085) and OS (HR 1.70, *unadjusted p* = 0.045, *adjusted p* = 0.181) in the MIBC subgroup after multivariate adjustment. Transcriptomic analysis suggested that MIBC patients with high UP-B grade may exhibit a more aggressive tumor phenotype.

**Conclusions:**

UPSQ was linked to the clinical outcomes of patients receiving PD-1-based immunochemotherapy for BC. In MIBC, lower baseline UPSQ grade may be associated with a tumor transcriptomic phenotype contributing to better cCR and OS.

**Clinical Trial Registration:**

https://clinicaltrials.gov, identifier NCT04730219; https://clinicaltrials.gov, identifier NCT04730232.

## 1 Introduction

Bladder cancer (BC) has a high incidence rate globally, ranking 10th in terms of morbidity among all malignancies worldwide (1). In recent years, treatments based on immune checkpoint inhibitors (ICIs) have been increasingly used in the treatment of BC and have shown favorable efficacy (2–5). However, as outcomes remain heterogeneous among individuals, the exploration of multiple biomarkers is still essential. Biomarkers such as PD-1/PD-L1 expression, tumor mutational burden, circulating tumor DNA (ctDNA), and urinary tumor DNA (UtDNA) have been explored for their ability to predict or monitor outcomes in the setting of disease management (6–8). Among these approaches, non-invasive liquid biopsy, including ctDNA and UtDNA, are being validated for its effectiveness in outcomes prediction (7–9). However, the high cost of these tests limits their use for high-frequency monitoring. Thus, a low-cost, convenient, and rapid non-invasive assay remains an unmet clinical need as a complementary tool. Urinary protein semi-quantification (UPSQ) in urinalysis is widely used in clinical practice as a convenient, low-cost, non-invasive laboratory test to evaluate the concentration of urinary protein. The quantity of urinary protein has been previously proven to be correlated to the tumor volume load of urothelial carcinoma (10, 11). Nevertheless, the association between the UPSQ and clinical outcomes in the set of PD-1 immune checkpoint inhibitor-based immunochemotherapy (PD-1 based immunochemotherapy) for BC remains unverified.

The phase II TRUCE-01 (NCT04730219) and TRUCE-02 (NCT04730232) studies evaluated the efficacy of tislelizumab (a kind of programmed death-1 ICIs) plus nab-paclitaxel for localized muscle-invasive bladder cancer (MIBC) and very high-risk non-muscle-invasive bladder cancer (VHR-NMIBC) (12, 13), respectively. To reveal the correlation between UPSQ and outcomes in the set of PD-1-based immunochemotherapy for BC, we designed this *post-hoc* study based on TRUCE-01 and TRUCE-02 trials.

## 2 Material and methods

### 2.1 Study design and patient inclusion

**Figure 1:**
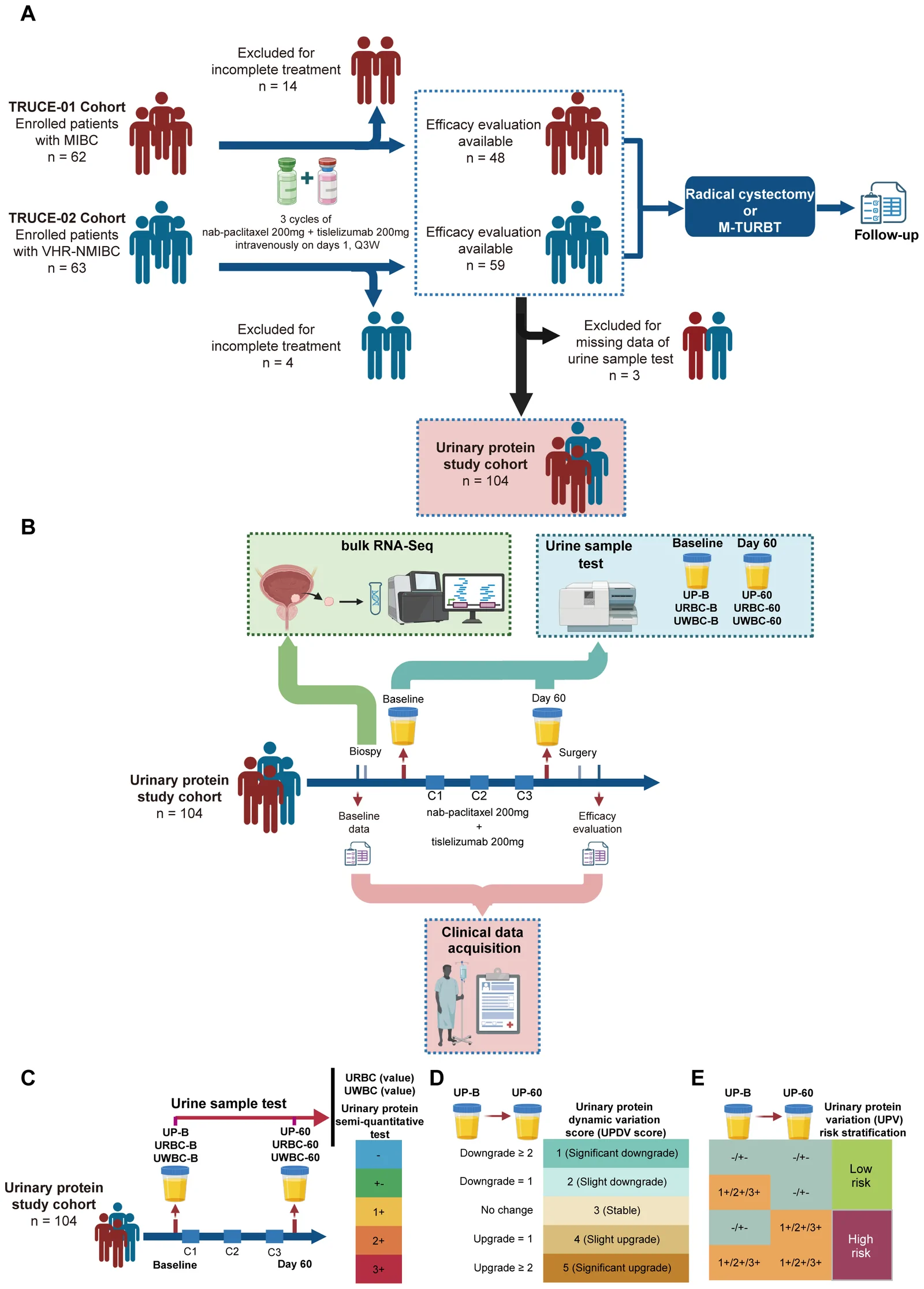
Schematic overview of the study design. **(A)** Flowchart of the study cohort enrollment. **(B)** Acquisition of tumor specimens, urine sample and clinical data. **(C)** Definitions of urinary indicators. **(D)** Definition of urinary protein dynamic variation score (UPDV score). **(E)** Definition of urinary protein variation risk stratification (UPV risk stratification). UP-B, urinary protein semi-quantification at baseline; UP-60, urinary protein semi-quantification at 60 days. The original components of this figure were created using BioRender.

This study relies on the population of the TRUCE-01 and TRUCE-02 trials. An evaluable population consisting of 48 patients with localized MIBC from TRUCE-01 study and 59 patients with localized VHR-NMIBC from TRUCE-02 study were included in the study. 3 patients were excluded for missing data of urine sample test, and 104 patients were preserved in the study (**Figure 1A**). All patients had undergone at least 3 cycles of tislelizumab (200mg) in combination with nab-paclitaxel (200mg) iv q3W and completed radical cystectomy (RC) or maximal transurethral resection of bladder tumor (m-TURBT) (**Figure 1B**).

### 2.2 Clinical data collection

Clinical data were collected from the central repository of the Second Hospital of Tianjin Medical University Healthcare System, including baseline demographic data, comorbidity (including obesity, hypertension, diabetes, and coronary artery disease (CAD)), smoking history, renal function (including estimated glomerular filtration rate (eGFR)), tumor size at baseline and post-treatment, and efficacy evaluation data (**Figure 1B**). The variation of tumor size (ΔTumor size) was defined as the change of tumor size from baseline to post-treatment (post-treatment tumor size – baseline tumor size). Follow-up information was last updated in May 2026.

### 2.3 Urine sample test

Urinalysis was conducted at baseline and 60 ± 7 days after treatment initiation, including UPSQ, urinary white blood cell count (in high magnification view) (UWBC) and red blood cell count (in high magnification view) (URBC) (**Figure 1B**). UPSQ/URBC/UWBC at baseline were named as UP-B/URBC-B/UWBC-B, and as UP-60/URBC-60/UWBC-60 at 60 ± 7 days, respectively (**Figure 1C**). To analyze the dynamic trend of UPSQ, we defined the urinary protein dynamic variation score (UPDV score, defined as the variation of UPSQ grade from baseline to 60 ± 7 days, and scores of 1 to 5 respectively represent “downgrade ≥ 2 levels”, “downgrade = 1 level”, “no change”, “upgrade = 1 level”, and “upgrade ≥ 2 levels”.) and urinary protein variation risk stratification (UPV risk stratification, defined as the risk pattern of the change between positive (1+/2+/3+) and non-positive (-/+-) UPSQ before and after treatment. “Low risk” is defined as a continuous “-/+-” state or a transition from the “1+/2+/3+” state to the “-/+-” state, while “high risk” is defined as a continuous “1+/2+/3+” state or a transition from the “-/+-” state to the “1+/2+/3+” state.) (details shown in **Figures 1D, E**). Moreover, to measure the changes of URBC and UWBC, we defined the variation of URBC [ΔURBC, defined as (URBC-60 – URBC-B)],variation of UWBC [ΔUWBC, defined as (UWBC-60 – UWBC-B)], variation rate of URBC [RΔURBC, defined as (URBC-60 – URBC-B)/(URBC-B)], and variation rate of UWBC [RΔUWBC, defined as (UWBC-60 – UWBC-B)/(UWBC-B)].

### 2.4 RNA-seq and transcriptomic analyses

Baseline tumor specimens from 22 MIBC patients were obtained via cystoscopic biopsy. Total RNA was extracted using RNeasy Mini Kit (Qiagen, Valencia, CA). Sequencing libraries were prepared with the NEBNext Ultra™ RNA Library Prep Kit for Illumina (NEB, USA) following the manufacturer’s instructions. The libraries were then sequenced on an Illumina HiSeq platform to generate 125–150 bp paired-end reads. Raw reads were aligned to the human reference genome GRCh38 using HISAT2, and gene expression levels were quantified with default parameters.

### 2.5 Study endpoints

The primary endpoint is clinical complete response (cCR), defined as the absence of localize lesions on imaging evaluation, negative urine cytology, and pathological confirmation of no residual tumor via RC or m-TURBT. The secondary endpoints include overall survival (OS, defined as the time from treatment initiation to death from any cause), cancer-specific survival (CSS, defined as the time from treatment initiation to death caused by the bladder cancer) and event-free survival (EFS, defined as the time from treatment initiation to disease progression or death from any cause).

### 2.6 Statistical analysis

For characteristics at baseline, categorical variables were expressed as frequencies and percentages, continuous variables with normal distribution were presented as means ± standard deviations (SD), and non-normally distributed variables were reported as medians and interquartile ranges (IQR). The values of URBC and UWBC were log10(x + 1)-transformed to reduce data dispersion. The variation among UPSQ, URBC, and UWBC was evaluated through paired Wilcoxon signed-rank tests. Correlation analyses were carried out using the Spearman correlation test. Logistic regression was conducted to identify the factors related to cCR. The trend of cCR rate among UPSQ grades/UPDV scores was evaluated by Cochran-Armitage trend tests (CAT test). Survival outcomes were evaluated using Kaplan-Meier analysis and Cox regression. Benjamini-Hochberg (BH) correction was applied in regression analyses. All statistical analyses and visualizations were performed using SPSS 26.0 and R 4.5.2.

## 3 Results

### 3.1 Patient characteristics

Baseline characteristics of 104 patients are shown in **Table 1**, with 47 (45%) cases with MIBC and 57 (55%) with VHR-NMIBC. A total of 85 (82%) males and 19 (18%) females enrolled in our study. At baseline, UPSQ of 36 (35%) cases were scored as negative (-), 17 (16%) as trace (+-), 25 (24%) as mildly positive (1+), 17 (16%) as moderately positive (2+), and 9 (9%) as severely positive (3+).

**Table 1:** Baseline characteristics of patients.

Characteristics	Classifications	Overall	MIBC	VHR-NMIBC
n		104	47	57
Gender (%)	Female	19 (18.3)	10 (21.3)	9 (15.8)
	Male	85 (81.7)	37 (78.7)	48 (84.2)
Age (mean (SD))		66.6 (8.4)	67.4 (8.9)	65.9 (8.0)
Hydronephosis (%)	No	87 (83.7)	36 (76.6)	51 (89.5)
	Yes	17 (16.3)	11 (23.4)	6 (10.5)
Hypertension (%)	No	42 (40.4)	18 (38.3)	24 (42.1)
	Yes	62 (59.6)	29 (61.7)	33 (57.9)
Diabetes (%)	No	83 (79.8)	39 (83.0)	44 (77.2)
	Yes	21 (20.2)	8 (17.0)	13 (22.8)
CAD* (%)	No	87 (83.7)	40 (85.1)	47 (82.5)
	Yes	17 (16.3)	7 (14.9)	10 (17.5)
Obesity (%)	No	94 (90.4)	44 (93.6)	50 (87.7)
	Yes	10 (9.6)	3 (6.4)	7 (12.3)
History of Smoking (%)	No	46 (44.2)	20 (42.6)	26 (45.6)
	Yes	58 (55.8)	27 (57.4)	31 (54.4)
UWBC* (median [IQR])		18.5 [8.1, 56.6]	11.9 [4.8, 45.7]	22.2 [11.4, 62.7]
URBC* (median [IQR])		74.8 [15.8, 260.3]	37.9 [10.9, 254.5]	104.5 [30.5, 260.1]
UPSQ* (%)	–	36 (34.6)	23 (48.9)	13 (22.8)
	+-	17 (16.3)	4 (8.5)	13 (22.8)
	1+	25 (24.0)	11 (23.4)	14 (24.6)
	2+	17 (16.3)	6 (12.8)	11 (19.3)
	3+	9 (8.7)	3 (6.4)	6 (10.5)
eGFR (median [IQR])		87.0 [75.9, 95.6]	82.5 [70.0, 94.9]	88.4 [80.7, 97.1]

*CAD, Coronary Artery Disease; UWBC, urinary white blood cell count (in high magnification view); URBC, urinary red blood cell count (in high magnification view); UPSQ, Urinary protein semi-quantification; eGFR, estimated glomerular filtration rate.

### 3.2 Patterns of UPSQ from baseline to day 60

**Figure 2:**
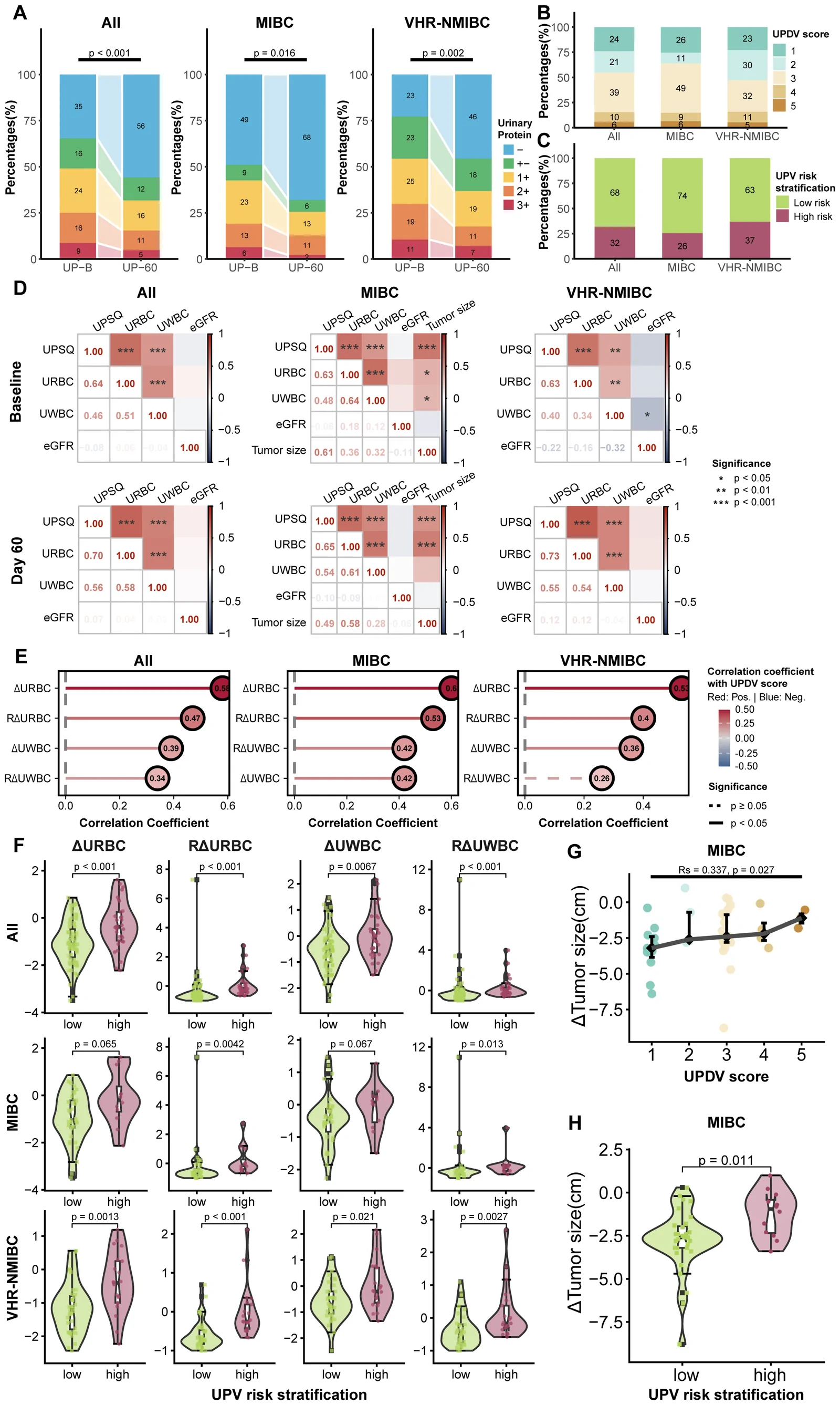
Patterns of UPSQ during the immunochemotherapy. **(A)** Variation of UPSQ grade from baseline to day 60 in all study cohort, MIBC cohort and VHR-NMIBC cohort. **(B)** Proportions of UPDV scores in all study cohort, MIBC cohort and VHR-NMIBC cohort. **(C)** Proportions of UPV risk stratification in all study cohort, MIBC cohort and VHR-NMIBC cohort. **(D)** Correlation among UPSQ, URBC, UWBC, eGFR in all study cohort, MIBC cohort and VHR-NMIBC cohort at baseline and day 60. In MIBC cohort, correlation between tumor size and other indicators were also shown. **(E)** Correlation between UPDV score and ΔURBC/RΔURBC/ΔUWBC/RΔUWBC in all study cohort, MIBC cohort and VHR-NMIBC cohort. **(F)** Comparison of ΔURBC/RΔURBC/ΔUWBC/RΔUWBC between patients with UPV low risk and high risk in all study cohort, MIBC cohort and VHR-NMIBC cohort. **(G)** Correlation between UPDV score and ΔTumor size in MIBC cohort. **(H)** Comparison of ΔTumor size between patients with UPV low risk and high risk in MIBC cohort. UPDV score, urinary protein dynamic variation score; UPV risk stratification, urinary protein variation; UWBC, urinary white blood cell count (in high magnification view); UPSQ, urinary protein semi-quantification; eGFR, estimated glomerular filtration rate; MIBC, muscle-invasive bladder cancer; VHR-NMIBC, very high risk non-muscle-invasive bladder cancer; ΔURBC, variation of URBC; ΔUWBC, variation of UWBC; RΔURBC, variation rate of URBC; RΔUWBC, variation rate of UWBC.

We compared UP-B and UP-60 measured in each patient and presented the results in **Figure 2A**. Compared to UP-B, UP-60 showed a trend of downgrade in all cohort, MIBC subgroup and VHR-NMIBC subgroup (All, MIBC, VHR-NMIBC: *p* < 0.001, *p* = 0.016, *p* = 0.002). 84%, 85%, and 84% of the UPDV scores were ≤ 3, and 68%, 74%, and 63% of the UPV risk stratifications were low risk in patients across all cohorts, the MIBC subgroup, and the VHR-NMIBC subgroup, respectively (**Figures 2B, C**). To explore the factors influencing the UPSQ, we performed the correlation analysis, indicating that URBC and UWBC are significantly positively correlated with UPSQ, whether at baseline or day 60 of treatment (**Figure 2D**). Correlation between dynamic of UPSQ and URBC/UWBC also explored in the study. In all cohort, MIBC subgroup, and VHR-NMIBC subgroup, we found ΔURBC, ΔUWBC, RΔURBC, and RΔURBC were positively related with UPDV score (**Figure 2E**). In patients with UPV high risk, ΔURBC, ΔUWBC, RΔURBC, and RΔURBC were higher than patients with UPV low risk (**Figure 2F**). Interestingly, in the subgroup of MIBC, the tumor size and UPSQ showed a significant positive correlation (**Figure 2D**). Similar trend was also found between ΔTumor size and UPDV score (Rs = 0.337, *p* = 0.027) (**Figure 2G**). In patients with UPV low risk, the trend of tumor shrinkage was more pronounced than patients with high risk (ΔTumor size, low risk vs. high risk: -2.60cm vs. -0.95cm, *p* = 0.011) (**Figure 2H**).

### 3.3 Correlation between UPSQ and clinical outcomes

#### 3.3.1 UPSQ and cCR

**Figure 3:**
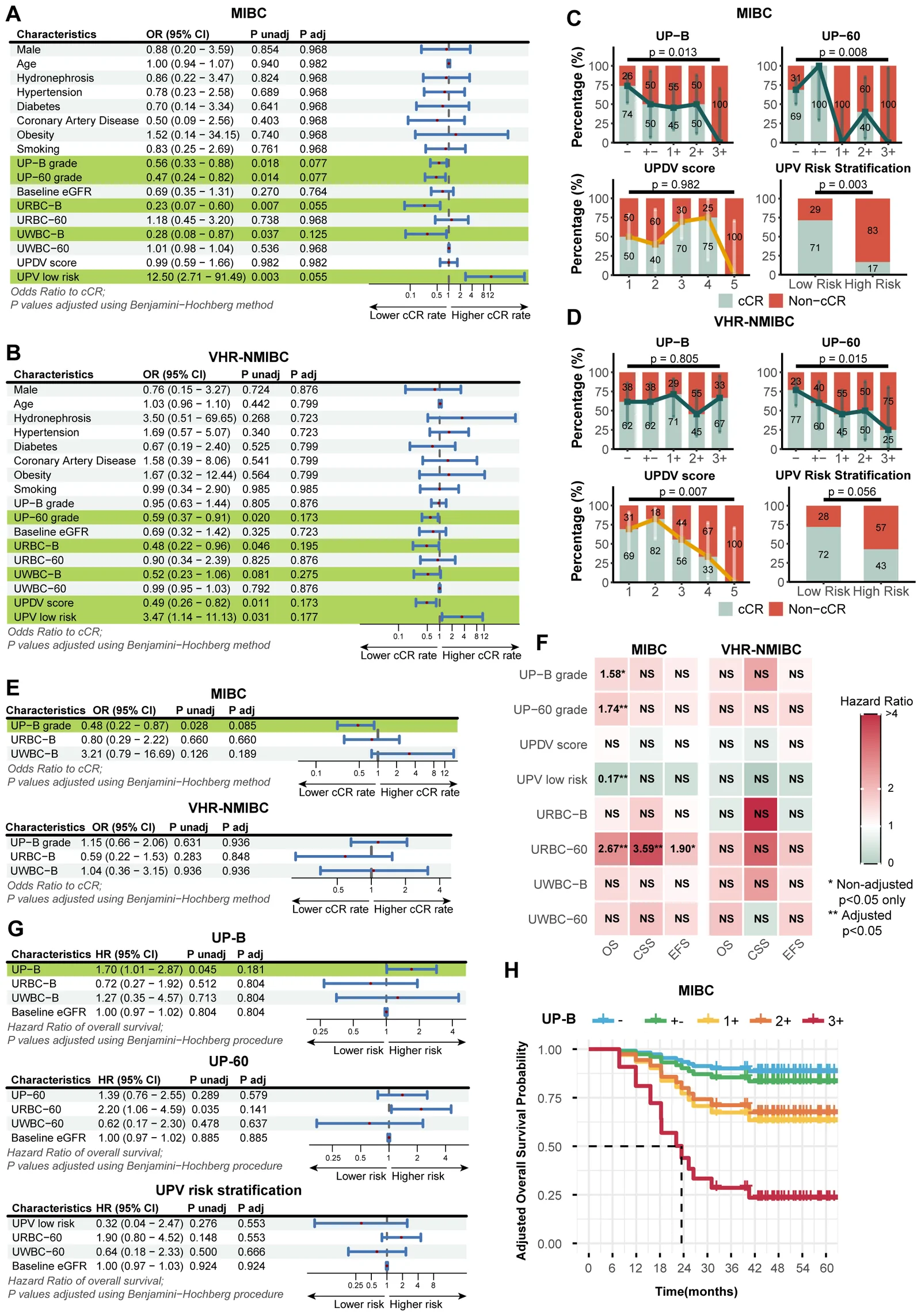
Univariable and multivariable models reveal the correlation between UPSQ and clinical outcomes in MIBC and VHR-NMIBC cohort. **(A)** Univariate logistic regression of factors associated with cCR in the MIBC cohort. **(B)** Univariate logistic regression of factors associated with cCR in the VHR-NMIBC cohort. **(C)** Stacked bar chart illustrating cCR rates stratified by UP-B, UP-60, UPDV score, and UPV risk stratification in the MIBC cohort. **(D)** Stacked bar chart illustrating cCR rates stratified by UP-B, UP-60, UPDV score, and UPV risk stratification in VHR-NMIBC cohort. **(E)** Multivariable logistic regression forest plot showing adjusted odds ratios for cCR associated with UP-B, URBC-B, and UWBC-B in the MIBC and VHR-NMIBC cohorts. **(F)** Univariate Cox regression of urinary indicators associated with survival (including OS, CSS and EFS) in the MIBC and VHR-NMIBC cohorts. **(G)** Multivariable-adjusted (by URBC, UWBC and baseline eGFR) predictive performance of UP-B, UP-60 and UPV risk stratification for OS in the MIBC cohort. **(H)** Adjusted OS curves by UP-B grade, derived from the multivariable Cox regression model. UP-B, urinary protein semi-quantification at baseline; UP-60, urinary protein semi-quantification at 60 days; UPDV score, urinary protein dynamic variation score; UPV risk stratification, urinary protein variation; UWBC, urinary white blood cell count (in high magnification view); UPSQ, urinary protein semi-quantification; eGFR, estimated glomerular filtration rate; MIBC, muscle-invasive bladder cancer; VHR-NMIBC, very high risk non-muscle-invasive bladder cancer; P unadj, Unadjusted p value; P adj, Adjusted p value.

All 104 patients in our study have completed at least 3 cycles of treatment and underwent efficacy evaluations. 62 (60%) out of 104 cases achieved cCR, while 27 were MIBC, and 35 cases were VHR-NMIBC. In regression analyses, several indicators reached nominal significance (*unadjusted p* < 0.05); however, these associations did not remain statistically significant (*adjusted p* > 0.05) after BH correction due to limited sample size. In MIBC subgroup, UPV low risk was associated with a higher cCR rate (OR 12.5, 95% CI 2.71-91.49, *unadjusted p* = 0.003, *adjusted p* = 0.055), while higher UP-B grade, UP-60 grade, URBC-B value and UWBC-B value exhibited a lower cCR rate, though the difference was not statistically robust after adjustment (UP-B grade OR 0.56, 95% CI 0.33-0.88, *unadjusted p* = 0.018, *adjusted p* = 0.077; UP-60 grade OR 0.47 95% CI 0.24-0.82, *unadjusted p* = 0.014, *adjusted p* = 0.077; URBC-B OR 0.23, 95% CI 0.07-0.60, *unadjusted p* = 0.007, *adjusted p* = 0.055; UWBC-B OR 0.28, 95% CI 0.08-0.87, *unadjusted p* = 0.037, *adjusted p* = 0.125) (**Figure 3A**). In VHR-NMIBC subgroup, UPV low risk was also associated with a higher cCR rate (OR 3.47, 95%CI 1.14-11.13, *unadjusted p* = 0.031, *adjusted p* = 0.177), and higher UP-60 grade, URBC-B value and UPDV score were related to lower cCR rate (UP-60 grade OR 0.59 95% CI 0.37-0.91, *unadjusted p* = 0.020, *adjusted p* = 0.173; URBC-B OR 0.48, 95%CI 0.22-0.96, *unadjusted p* = 0.046, *adjusted p* = 0.195; UPDV score OR 0.49, 95% CI 0.26-0.82, *unadjusted p* = 0.011, *adjusted p* = 0.275) (**Figure 3B**). The trend of cCR rate with UP-B, UP-60, UPDV score and UPV risk stratification was shown in **Figures 3C, D**. Other clinical factors, such as gender, age, and comorbidities, were not significant in the univariate model. To further explore the potential independent association of UP-B grade with cCR while accounting for the influence of URBC and UWBC, we included UP-B together with URBC-B and UWBC-B in a multivariable model. In the MIBC subgroup, UP-B grade exhibited a trend toward a lower cCR rate, although this association did not remain significant after FDR correction (OR 0.48, 95% CI 0.22-0.87, *unadjusted p* = 0.028, *adjusted p* = 0.085) (**Figure 3E**).

#### 3.3.2 Correlation between UPSQ and survival

The median duration of follow-up is 44.5 months (range 4.8 to 63.3). Events of OS, CSS and EFS occurred in 15 (14%), 7 (7%) and 21 (20.1%) patients, respectively. Median OS, CSS, and EFS were not reached. Univariable Cox regression analyses were conducted in the MIBC and VHR-NMIBC cohort, respectively (**Figure 3F**; **Supplementary Table 1**), indicating that in the MIBC cohort, higher UP-60 grades correlated significantly to a poorer OS, whereas UPV low risk was linked to a better OS (UP-60: HR 1.74, 95% CI 1.16-2.60, *unadjusted p* = 0.007, *adjusted p* = 0.040; UPV low risk: HR 0.17, 95% CI 0.05-0.57, *unadjusted p* = 0.004, *adjusted p* = 0.034). Moreover, higher UP-B showed nominal associations with poorer OS, but this association did not remain statistically significant after adjustment (HR 1.58, 95% CI 1.02-2.43, *unadjusted p* = 0.039, *adjusted p* = 0.166) (**Figure 3F**). Further, we conducted the multivariable analyses adjusting for URBC-B, UWBC-B and baseline eGFR and found that UP-B grade exhibited a trend toward worse OS, although this association did not survive after BH correction (HR 1.70, 95% CI 1.01-2.87, *unadjusted p* = 0.045, *adjusted p* = 0.181). In contrast, UP-60 and UPV low risk were not retained in the multivariable models (**Figure 3G**). Interestingly, at 60 days, higher URBC-60, but not UP-60 grade, was related to worse OS in multivariable models (**Figure 3G**). The adjusted OS survival curves by UP-B grade, derived from the multivariable Cox regression model, are presented in **Figure 3H**. No significant correlation between survival and urine indicators was found in the VHR-NMIBC cohort (**Figure 3F**).

### 3.4 Transcriptomic phenotype and UP-B grade in patients with MIBC

**Figure 4:**
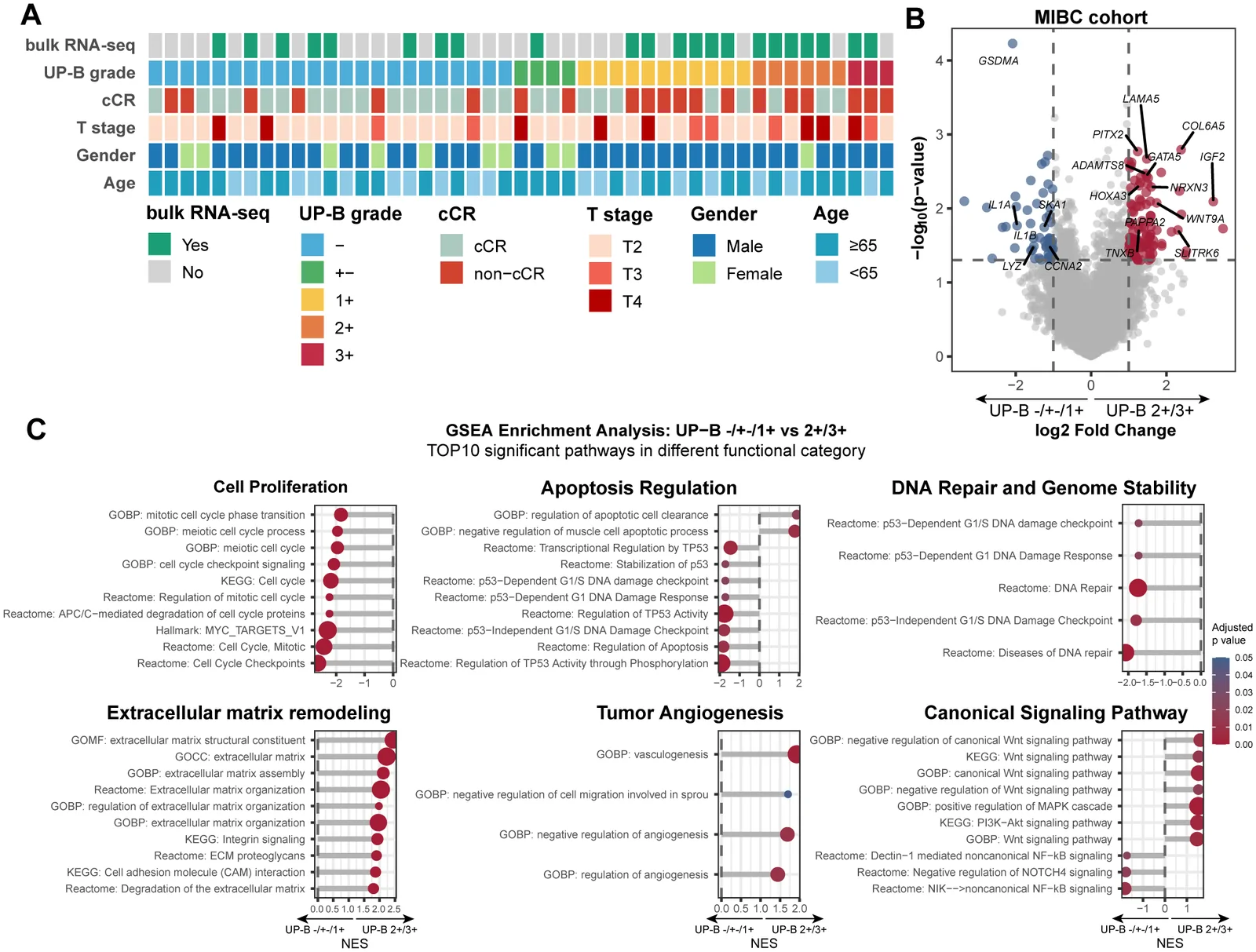
Bulk RNA-seq revealed different transcriptomic phenotypes between high UP-B and low UP-B grade tumor in MIBC cohort. **(A)** Overview of bulk RNA-seq data acquisition, UP-B grade, cCR occurrence and clinical features in the MIBC cohort. **(B)** Volcano plot of differential expression analysis between high UP-B (2+/3+) group and low UP-B (-/+-/1+) group. **(C)** GSEA enrichment analysis comparing high UP-B group and low UP-B group. UP-B = urinary protein semi-quantification at baseline; UP-60 = urinary protein semi-quantification at 60 days.

Transcriptomic profiling was performed on untreated tumors from 22 patients with MIBC to explore the transcriptomic phenotype among tumor with different UP-B grade. (**Figure 4A**). We defined -, +- and 1+ as low UP-B grade, while 2+ and 3+ as high UP-B grade. Compared to patients with high UP-B grade, tumor from patients with low UP-B grade showed a trend toward higher expression of genes known to be related to extracellular matrix remodeling (for example, LAMA5 (14)) and angiogenesis (for example, IGF2 (15)) (**Figure 4B**). However, no gene remained statistically significant after FDR correction due to the limited sample size.

Gene set enrichment analysis (GSEA) (16, 17) using gene sets from the Gene Ontology (GO) (18), Kyoto Encyclopedia of Genes and Genomes (KEGG) (19), Reactome (20), and Hallmark (21) databases indicated that, in the low UP-B group, gene sets associated with cell proliferation, apoptosis regulation and DNA repair were significantly enriched (**Figure 4C**). On the contrary, tumors in the high UP-B group were upregulated in the extracellular matrix remodeling pathway and tumor angiogenesis pathway (**Figure 4C**). Moreover, in the high UP-B group, several canonical signaling pathways, such as the Wnt signaling pathway, PI3K-Akt signaling pathway and MAPK pathway were enriched, while the noncanonical NF-kB pathway was repressed (**Figure 4C**). Owing to the small sample size, these results must be viewed with caution and require validation in larger-scale studies.

Immune infiltration analyses were conducted by using CIBERSORT (22), Xcell (23) and TcellSI (24). Owing to the limited sample size, most immune infiltration scores did not show a significant correlation with UP-B grade, and they should be interpreted with extreme caution. Cancer-associated fibroblast (CAF) infiltration showed a positive trend (Rs = 0.39, *p* = 0.07), though statistical significance was not reached. Furthermore, based on the TCellSI score, infiltration of T cells in quiescence status tended to be positively correlated with UP-B (Rs = 0.36, *p* = 0.10), while proliferating T cells exhibited a trend toward a negative correlation with UP-B (Rs = -0.14, *p* = 0.34) (**Supplementary Figure 1**).

## 4 Discussion

Our study evaluated the UPSQ at baseline and 60 days after treatment initiation and its dynamic patterns in the MIBC cohort and VHR-NMIBC cohort, exploring the correlation between UPSQ and clinical outcomes in the set of PD-1-based immunochemotherapy for BC. In the MIBC cohort, UP-B grade showed an inverse association with cCR and OS. However, these associations did not remain statistically significant after BH correction. Transcriptomic analysis indicated that different UP-B grades may be linked to distinct tumor molecular phenotypes and immune microenvironment characteristics. As a simple, non-invasive laboratory test, UPSQ may serve as a potential biomarker of cCR and OS in the set of immunochemotherapy for MIBC, but its clinical applicability requires validation in independent, larger cohorts.

From UP-B to UP-60, the downgrade of UPSQ was common in both MIBC and VHR-NMIBC cohorts, indicating the mechanism underlying the occurrence of urinary protein was modulated by immunochemotherapy. The sources of protein in the urine of patients with BC are complex, which may originate from tumor vessels leakiness (25, 26), urinary tract infections (UTI) (27), tumor-derived secretions (28, 29), and paraneoplastic nephropathy (10, 11, 30, 31). Larger bladder tumors contribute to elevated UPSQ through multiple factors: more tumor vessels, higher risk of UTI (27), more release of tumor-derived secretory proteins (28, 29), and more kidney-derived protein release caused by tumor-associated immune complexes driving paraneoplastic nephropathy (11). In this study, we found that the UPSQ correlated with URBC and UWBC. Notably, tumor volume of MIBC showed consistently positive correlations with all three parameters, indicating that tumor enlargement may be the initial trigger for URBC and UWBC elevation, which is subsequently related to the rise in UPSQ. Hemmingsen et al. found that an obvious reduction in urinary protein has been observed in patients responding to antitumor treatment (32). Accordingly, we propose that in patients responding to PD-1 immunochemotherapy, tumor shrinkage leads to a significant decline in UPSQ. Indeed, validating this hypothesis in animal models in the future is essential.

Notably, in the MIBC cohort, higher UP-B grade exhibited a trend toward a lower cCR rate and worse OS in both univariable and multivariable models, but failed to remain significant after BH correction. To our knowledge, this is the first study to explore the predictive value of UP-B for treatment response in MIBC patients receiving immunochemotherapy. On the contrary, patients in VHR-NMIBC showed no correlation between UP-B grade and clinical outcomes. In general, the tumor volume of NMIBC tends to be smaller than MIBC, which may make it difficult to distinguish real differences in urinary protein grade by UPSQ. Therefore, it is hard for UP-B grade to predict the clinical outcomes in VHR-NMIBC.

To further explore the mechanisms underlying the predictive value of UP-B grade for cCR and OS in MIBC, we collected and analyzed transcriptomic data from baseline tumor tissues of 22 MIBC patients and found that patients with higher UP-B grade may exhibit distinct molecular phenotypes compared with those with lower UP-B grade. In tumors with high UP-B grade, apoptosis regulation pathways, DNA repair pathways and cell proliferation pathways were down-regulated, whereas extracellular matrix remodeling, angiogenesis, and several canonical oncogenic cascades, including PI3K-Akt, MAPK, and Wnt signaling were up-regulated. These molecular features suggest that MIBC may be more aggressive with higher UP-B grade (33–41). Of course, due to the small sample size, these findings still require large-scale cohort studies for further validation.

To our knowledge, this is the first study to explore the correlation of UPSQ-clinical outcomes in the set of immunochemotherapy for BC. As a non-invasive, low-cost and convenient laboratory test, UPSQ can be repeatedly performed at every treatment cycle, making it uniquely suited for tumor dynamic monitoring during immunochemotherapy. Furthermore, in patients with MIBC, UPSQ appeared to be associated with transcriptomic and immune microenvironment patterns that may be linked to treatment response and survival. In recent years, a series of novel non-invasive liquid biopsy techniques, including UtDNA, ctDNA, and urine methylation analysis, have been validated for the diagnosis and outcomes prediction of BC (7–9, 42–48). Moreover, several studies successfully applied these approaches in prognostic monitoring and treatment decision-making in immunotherapy (49, 50). Given its low cost and convenience, UPSQ may serve as an ideal complement to these techniques, facilitating the future construction of multi-omics predictive models. Currently, our center is actively conducting research on urine-based liquid biopsy, and we aim to integrate these technologies to build multi-omics models for predicting immunotherapy efficacy and guiding treatment decisions in BC.

There are several limitations in our study: firstly, the sample size was small, limiting statistical power and the robustness of the reported associations. Therefore, validation conducted in a larger and multi-center cohort in the future is necessary, and such studies are currently in progress. Secondly, as a single-center study, the lack of available independent cohorts for external validation limited the accuracy and generalizability of its findings. Thus, further validation in independent, multicenter cohorts is essential, and now we are conducting multicenter, large-sample phase II clinical studies to complete this part of the work. Moreover, limited by small sample size, the results of transcriptomic analyses was intended solely as a preliminary exploration to provide clues for future research, and cannot serve as confirmatory evidence. Finally, UPSQ are influenced by confounding factors such as urinary tract infections, strenuous exercise, and postural changes. Although we have adjusted for UWBC and URBC, interference from non-tumor-related proteinuria cannot be fully excluded.

In conclusion, UPSQ was linked to the clinical outcomes of patients receiving PD-1 ICIs therapy for BC. In the MIBC cohort, lower baseline UPSQ grade may be associated with a tumor transcriptomic phenotype that contributes to more favorable cCR and OS.

## Data Availability

The datasets presented in this study can be found in online repositories. The names of the repository/repositories and accession number(s) can be found in the article/**Supplementary Material**.

## Supplementary material

Supplementary Figure 1 Correlation analysis between UP-B grade and immune infiltration scores.
